# EHMTI-0271. Refractory chronic daily headache and idiopathic intracranial hypertension: preliminary results of a prospective study

**DOI:** 10.1186/1129-2377-15-S1-D19

**Published:** 2014-09-18

**Authors:** V Favoni, F Toni, M Messia, C La Morgia, R Terlizzi, G Giannini, M Leonardi, P Cortelli, S Cevoli, G Pierangeli

**Affiliations:** 1 Department of Biomedical and NeuroMotor Sciences (DIBINEM) Alma Mater Studiorum-University of Bologna, IRCCS Scienze Neurologiche, Bologna, Italy; 2 Neuroradiology Department, IRCCS Scienze Neurologiche, Bologna, Italy; 3 IRCCS Scienze Neurologiche, IRCCS Scienze Neurologiche, Bologna, Italy

## Background

Recent evidences suggest an association between unresponsive chronic daily headache (CDH) and idiopathic intracranial hypertension without papilledema (IIHWOP). Diagnosis can be challenging. The CSF opening pressure (OP) cutoff value greater than 200 or 250 mmH2O is debated as the role of transverse sinus stenosis (TSS).

## Aim

To investigate the frequency of IIHWOP and TSS in adult patients with refractory CDH.

## Methods

In a prospective study, patients with refractory CDH were consecutively enrolled. Each participant underwent ophthalmologic evaluation and Optical Coherence Tomography to rule out the presence of papilledema; cerebral MR venography (MRV) to detect TSS; and a lumbar puncture in the lateral decubitus position to measure OP.

## Results

Among the 23 consecutive patients enrolled, 19 (13 F, 6 M; mean age 47.9 ± 12.3; mean BMI 26.1± 4.8) completed the study, 4 female patients dropped out. None of the 19 patients had papilledema. We found a TSS in 11 of 19 cases (58%): bilateral in 3 patients and unilateral in 8 cases. All of 19 cases displayed OP lower than 250 mmH2O (range 102-245) and normal CSF composition. We found a OP greater than 200 mmH2O only in four patients (1M, 3 F) (17%): two of them achieved an improvement of headache intensity and frequency after 12-18 ml CSF withdrawal; one of them had bilateral TSS.

## Conclusions

Our preliminary data suggest that IIHOWP may be an underestimated condition. We suggest to keep the 200 mm H2O cutoff value at least until the role of venous stenosis will be clarified.

No conflict of interest.

